# Optimization of echo-enabled harmonic generation toward coherent EUV and soft X-ray free-electron laser at NSLS-II

**DOI:** 10.1038/s41598-022-13702-3

**Published:** 2022-06-08

**Authors:** X. Yang, G. Penn, L. H. Yu, V. Smaluk, T. Shaftan

**Affiliations:** 1 National Synchrotron Light Source II, Brookhaven National Laboratory, Upton, NY 11973 USA; 2 Lawrence Berkeley National Laboratory, Berkeley, CA 94720 USA

**Keywords:** Free-electron lasers, High-harmonic generation

## Abstract

Prebunching via echo-enabled harmonic generation (EEHG) is an efficient way to reduce the radiator length and improve the longitudinal coherence as well as output stability in storage-ring-based free-electron lasers. We propose a conceptual design, which uses two straight sections to seed coherent extreme-ultraviolet (EUV) and soft X-ray emission with nearly MHz repetition rate. To take the large energy spread (10^−3^) of a storage ring into account and utilize the existing bending magnets between the two straight sections as the first chicane, we implement a special modeling tool, named EEHG optimizer. This tool has been successfully applied to maximize the prebunching with a reasonably low energy modulation, thereby generating intense coherent X-ray pulses within a short undulator length (a few meters) limited by the available space of a storage ring. Numerical simulations confirm that the optimized EEHG parameters can be directly applied to generate a 10 MW scale peak power with fully coherent ultrafast EUV to soft X-ray pulses based on the NSLS-II parameters. This method can be easily extended to other types of diffraction-limited storage rings.

## Introduction

Compared to conventional high-gain harmonic generation (HGHG)^1–4^, the echo-enabled harmonic generation (EEHG) scheme is significantly less sensitive to energy spread, which is typically large in storage rings. Recently, to overcome such large energy spread, the use of transverse gradient undulators (TGUs) has been proposed for a high-gain short wavelength FEL driven by a diffraction-limited synchrotron light source^5^; however, this approach requires bunches to be diverted into an additional ring with its own rf cavities. Since the EEHG seeding option requires no change of the storage ring lattice and is fully compatible with other beamlines, the compact design currently studied could enable synchrotron light source based free-electron lasers (FELs) to produce intense coherent radiation pulses with short durations^6–11^. Such fully coherent ultrafast photon pulses up to the carbon K-edge offers unique opportunities to conduct high resolution spectroscopy on organic materials that are important in environmental science, medicine, biology, and bio-renewable energy materials^12^. Also, extending the pump-probe approach known from ultraviolet/visible spectroscopy to shorter wavelengths allows detailed studies of excited-state dynamics in organic molecules or biomolecular structures on a nanosecond to femtosecond time scale. Optical pump soft X-ray probe spectroscopy is a relatively new approach to detect and characterize optically dark states in organic molecules, to explore exciton dynamics, or to observe transient charge transfer states. Recent developments on extreme-ultraviolet (EUV) and soft X-ray sources based on EEHG storage-ring FELs open new opportunities for studying excited-state dynamics in organic molecules, together with the tremendous increase of computing power, allows understanding the excited-stage behavior even of very complex organic molecules in more detail^13,14^.

Driven by those scientific applications, the implementations of EEHG at the NSLS-II and the future diffraction-limited upgrade (NSLS-IIU)^15^ are presently studied as an option to improve the longitudinal coherence and output stability, toward fully coherent storage ring-based FELs. To take the unique challenge and characteristic of storage-ring-based EEHG FELs into account, we have developed a modeling tool, named EEHG optimizer. This toolkit has been successfully applied to the NSLS-II storage ring with an up to two orders of magnitude improvement of the spectral brightness regarding the 12 nm wavelength. This scheme can be easily extended to any other type of 4th generation diffraction-limited storage ring^15–20^.

## Results

### Development of storage-ring-based EEHG optimizer

#### Tuning echo parameters

Starting with two seed lasers, we aim to produce a large harmonic through EEHG^21,22^. There are two modulation stages, each followed by a chicane or a dogleg^23^. These stages are labeled as 1 and 2. In a storage-ring-based EEHG seeding scheme, since the first chicane is determined by the bending magnets (BM) between two straight sections, it is convenient to take the momentum compaction $R_{56}$ as a fixed quantity^23^, denoted $R_{1}$. Here, the momentum compaction, defined as the derivative of normalized path length difference to normalized momentum ($\frac{p}{L}\frac{dL}{{dp}}$), is a measure of the momentum dependence of path length^23^. The convention used here is that a typical chicane of four bend magnets has a negative value of $R_{56}$. The second parameter to choose is the energy modulation of the first stage, $\eta_{M1}$. It may also be characterized by its value relative to the energy spread (see below). Fixing a different parameter such as the second $R_{56}$ involves coupling with other parameters and is difficult to select an optimal value without detailed analysis.

The output wavelength, $\lambda_{r}$, is also an important criterion since it can result from microbunching at that wavelength or a sub-harmonic. Together with the wavelengths of external lasers and thus the modulations, these choices will determine the parameters in stage 2, with two possible solutions (see “Appling optimizer to NSLS-II” section). Given the external laser wavelength $\lambda_{1}$ and $\lambda_{2}$, the output wavelength ideally has the form:

1 $$k_{r} = p \cdot k_{1} + m \cdot k_{2}$$

where $k_{1,2} = 2\pi /\lambda_{1,2}$, and *m* and *p* must be integers. Usually, we assume $\lambda_{1}$, $\lambda_{2}$, and thus $\lambda_{r}$ are integer harmonics of a single wavelength, although it may not matter too much in either way. This constrains the choices of possible output wavelengths. Often *m* is small, so $p \gg 1$ roughly determines the wavelength. We can treat *m* as an additional choice (or knob) related to the dominant wavelength of the bunched beam, which is close to $m \cdot \lambda_{r}$. The other main parameter is the energy spread, $\sigma_{\eta }$, which is fixed in a storage-ring-based FEL (e.g., 10^−3^ for NSLS-II) by the equilibrium between radiation damping and energy diffusion.

#### Appling optimizer to NSLS-II

Based upon the procedures described in “Method” section, we have implemented an EEHG optimizer for the purpose of tuning all important parameters toward the ideal performance of an EEHG beamline in a synchrotron light source.

**Figure 1:**
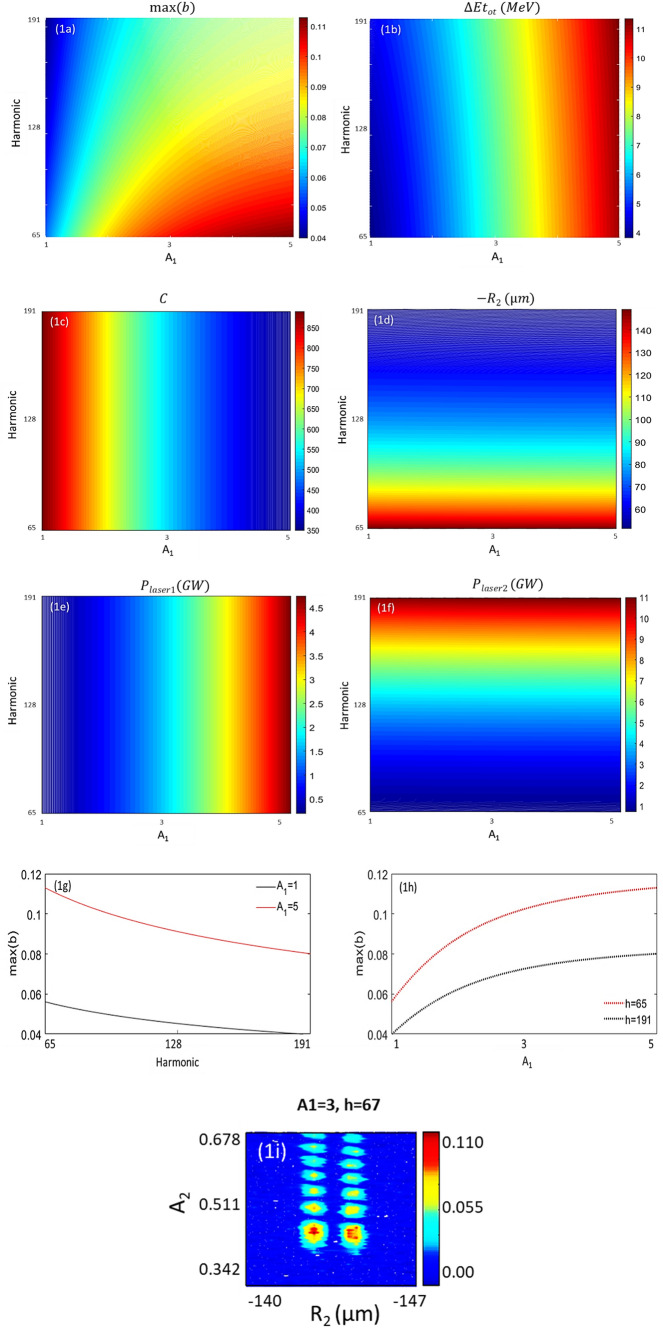
(**a**) The maximum bunching, (**b**) the final RMS energy spread, (**c**) the *C* parameter defined by Eq. 5 in “Method” section, (**d**) the optimal momentum compaction of chicane 2, (**e**) the powers of laser 1 and (**f**) laser 2 as functions of the harmonic (y axis) and the energy modulation of stage 1 (x axis) are shown as contours, respectively. *R*_2_ of chicane 2 should have the opposite sign relative to *R*_1_ of chicane 1, which is + 9.6 mm, hence, we plot the absolute value of *R*_2_. (**g**) Maximum bunching versus harmonic number is plotted for two cases: A_1_ = 1 (black) and A_1_ = 5 (red). (**h**) Maximum bunching versus modulation amplitude of stage 1 is plotted for two cases: harmonic 65 (red) and 191 (black). (**i**) With a fixed $A_{1} = 3$ and $R_{1} = + 9.6$ mm at harmonic 67, prebunching as functions of the energy modulation of stage 2 ($A_{2}$) and the momentum compaction of chicane 2 ($R_{2}$) is shown as the contour plot. It is evident that there are two optimal solutions with slightly different values of $R_{2}$ and $A_{2}$.

To cover the soft X-ray spectrum up to the carbon K-edge (4.13–12.4 nm), the harmonics of EEHG regarding the wavelength of stage 1, $\lambda_{1}$ = 800 nm, should be optimized in the range of 65–191. As example, we apply our newly developed storage-ring-based EEHG optimizer to the NSLS-II lattice. Since the EEHG layout will be discussed in “Desiging an EEHG FEL in a synchrotron light source” section with greater detail, we only show the maximum bunching (*b*), the final root mean square (RMS) energy spread (∆*E*_*tot*_), the *C* parameter (defined by Eq. 5 in “Method” section), the optimal momentum compaction of chicane 2 with an opposite sign (− *R*_2_), the powers of laser 1 (*P*_*laser1*_) and laser 2 (*P*_*laser2*_) as functions of the harmonic (y axis) and the energy modulation of stage 1 (x axis) as the contour plots in Fig. 1a–f, respectively. The *C* parameter decreases with the increase of the modulation amplitude and is about two orders of magnitude smaller than the two terms, $k_{r} R_{2}$ and $k_{1} R_{1}$, which are around 7.5 × 10^4^. The reasons why we choose the harmonic and the energy modulation of stage 1 as the input variables are:

- Once the harmonic, the momentum compaction of chicane 1 ($R_{1}$), and the energy modulation of stage 1 ($A_{1}$) are fixed, the Bessel functions with the maximal values of the bunching determine the optimal values of the momentum compaction of chicane 2 and the energy modulation of stage 2.
- The momentum compaction of chicane 1 is fixed by the momentum compaction of the storage ring lattice.
- The upper and lower limits of harmonics are determined by the user interested spectrum.
- The energy modulation of stage 1 must be optimized for maximum coherent radiation (CR) power. The goal can be achieved via maximizing the prebunching as well as mitigating the de-bunching effect through the radiator (see “Simulation for generating intense EUV and soft X-ray radiation” section for details).

Prebunching produced by EEHG is quite different from that produced from HGHG^1–4^. From harmonic 65 to harmonic 191, only about 30% decrease in the bunching factor is observed, as shown in Fig. 1g. There is still a significant amount of bunching (0.08) at harmonic 191 with $A_{1} = 5$. From Fig. 1h, we see that the maximal value of the bunching increases linearly with $A_{1}$ when $A_{1}$ is smaller than 2. When $A_{1}$ becomes larger than 3, the growth of maximal values slows down. This feature turns the storage-ring-based EEHG application into an attractive option via improving the longitudinal coherence with a moderate energy modulation. A small energy modulation is desired since the relative energy spread of a storage ring is a few to ten times larger compared to 10^−4^ often associated with linear-based accelerators. It is evident that there are two optimal solutions with the same bunching but slightly different values of $R_{2}$ and $A_{2}$, as shown in Fig. 1i.

### Desiging an EEHG FEL in a synchrotron light source

#### Design strategy

EEHG scheme enables the possibility for synchrotron light source based FELs to produce intense CR pulses with short durations. To achieve fully coherent storage ring-based FELs^15,24,25^, the implementations of EEHG at the NSLS-II and the future upgrade diffraction-limited NSLS-IIU are presently studied. We plan to use two straight sections for seeding the coherent EUV and soft X-ray emission at the repetition rate up to MHz.

There are a few important issues associated with the design of the EEHG beamline in the NSLS-II case^25^. They are:

- BM section between two straight sections provides the momentum compaction $R_{1}$ of the first chicane with + 9.6 mm.
- Modulation stage 1 and 2 must be separated by the BM section between the two straight sections.
- To maximize the CR power, the radiator needs to be positioned in the short straight section where the beta functions are small, hence the beam sizes are small.
- Large energy spread in RMS (10^−3^) limits the highest harmonic (e.g., 191 for carbon K-edge at 4.13 nm).
- The wavelength of laser 1 is fixed to 800 nm.
- The wavelength of laser 2 (λ_2_ = 400 nm) is chosen to be the second harmonic of laser 1.
- Selecting different parts of an electron bunch may extend the repetition rate from ~ 10 kHz up to 1 MHz.

Comparing λ_2_ = 400 nm with λ_2_ = 800 nm, the modulation amplitude of stage 2 can be reduced to half while keeping the same bunching factor regarding a specific harmonic. This can greatly benefit prebunching at high harmonics, with less energy modulation, thus, less beam heating. Also, repetition rate can surpass the limit that is set by the radiation damping time of a storage ring, ~ 10 ms for the NSLS-II^25^. The original 10 kHz repetition rate (1/10 ms × 100 bunches/turn) can be increased by a factor up to 100, which is approximately determined by the ratio of the electron bunch length and the modulated slice duration.

For this wavelength range, wake fields and stochastic energy scatter should not significantly impact the achieved bunching parameter.

#### Design of EEHG beamline

**Figure 2:**
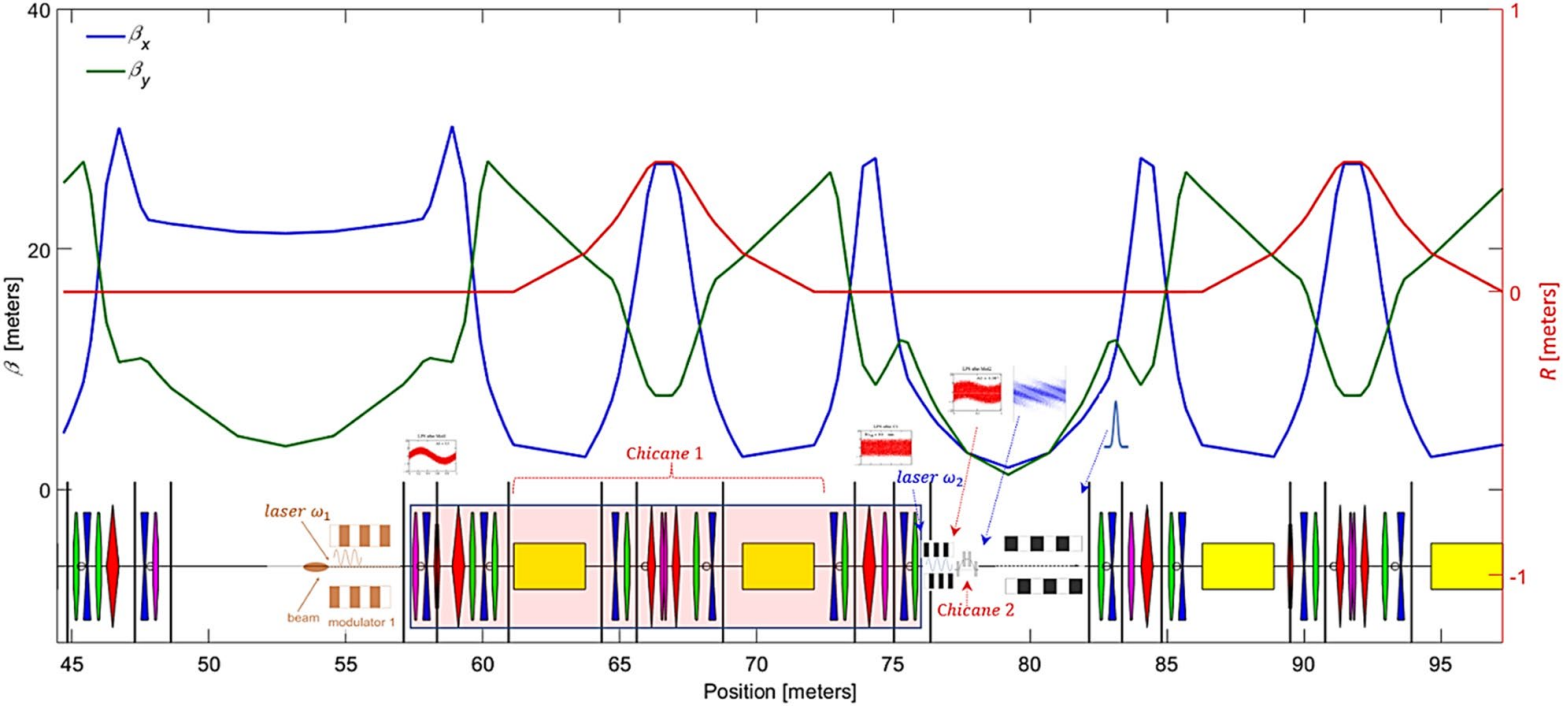
Schematic layout of EEHG at NSLS-II. Horizontal and vertical beta functions are plotted as the blue and the green curves, respectively. Horizontal dispersion is plotted as the red curve of the secondary y axis.

A schematic layout of the EEHG beamline at the NSLS-II storage ring is shown in Fig. 2. Stage 1, including modulator 1 and laser 1, is positioned downstream of the long straight section. Instead, stage 2, including modulator 2, laser 2, and chicane 2, locates upstream of the short straight section. The radiator is positioned downstream of the short straight section. The resonant wavelengths for modulator 1 and modulator 2 are 800 nm and 400 nm, respectively. The resonant wavelength of the radiator must cover the entire spectral range up to the carbon K-edge, from 4.13 to 12.4 nm, via varying the undulator gap.

The required seed laser power scales as the square of energy modulation amplitude and beam size, and the inverse square of undulator *K* parameter and length. To minimize the seed laser power of stage 2, the modulator 2 will be placed in the short straight section where the beta functions are small. The short straight section has a limited usable space, around 5 m, and will be occupied by three main elements of the EEHG beamline, including modulator 2, chicane 2, and the radiator. The magnetic field as a function of the undulator gap for a permanent magnet undulator (PMU) is given by

2 $$B_{0} \left( {gap,\lambda_{u} ,B_{max} ,M,h_{B} } \right) = 2 \cdot B_{max} \cdot \frac{M}{\pi } \cdot sin\left( {\frac{\pi }{M}} \right) \cdot \left( {1 - e^{{\frac{{ - 2 \cdot \pi \cdot h_{B} }}{{\lambda_{u} }}}} } \right) \cdot e^{{\frac{ - \pi \cdot gap}{{\lambda_{u} }}}} ,$$

where $M$ = 4 is the number of magnets per period, $h_{B} \left( {5\,{\text{cm}}} \right)$ is the height of magnets, and $B_{max}$ is the maximum *B* field^26^. The *K* parameter as a function of the gap is given by

3 $$K\left( {gap, \lambda_{u} , B_{max} } \right) = \frac{{B_{0} \left( {gap, \lambda_{u} , B_{max} , M, h_{B} } \right) \cdot \left( {e_{0} \cdot \lambda_{u} } \right)}}{{2 \cdot \pi \cdot (m_{0} c)}}.$$

**Figure 3:**
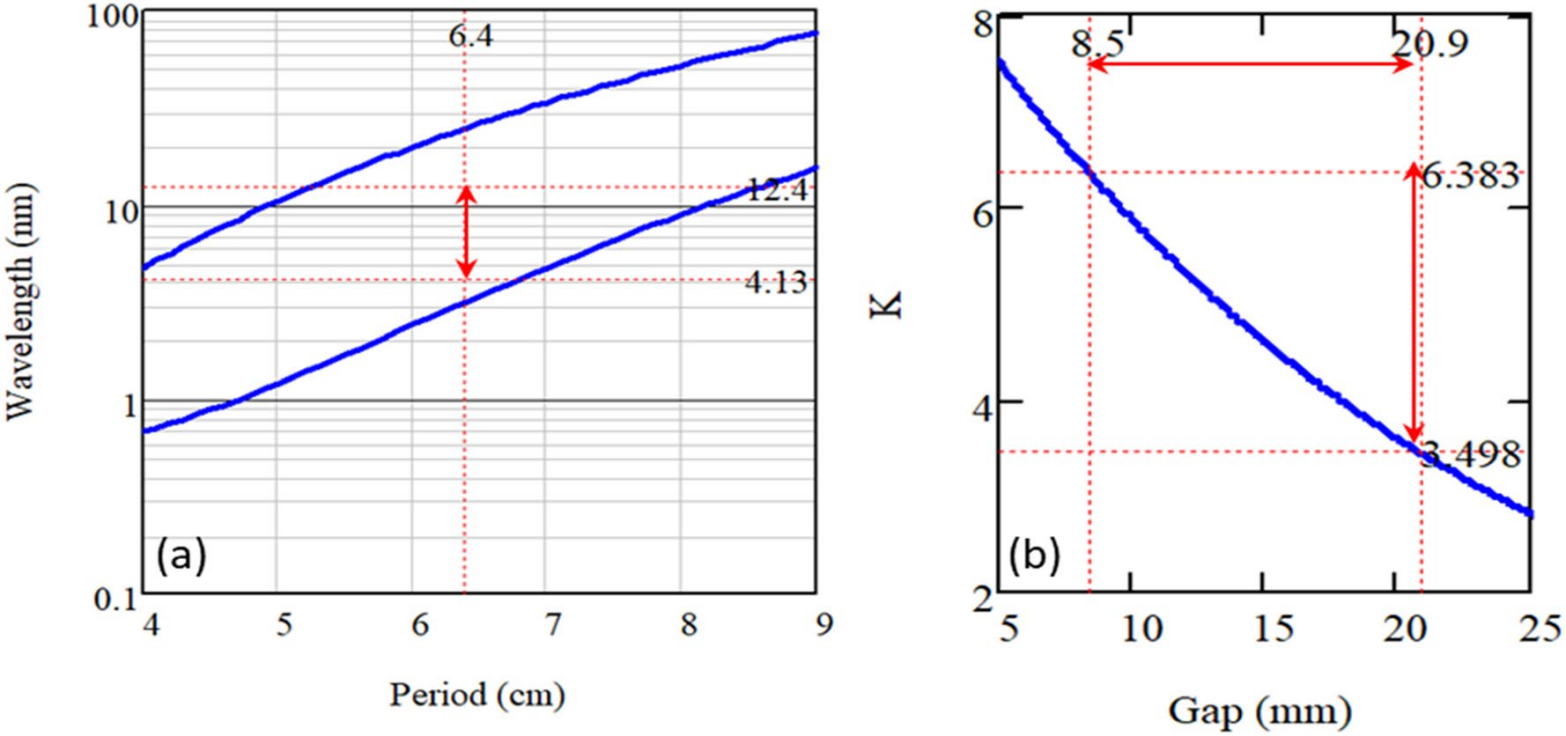
For the radiator with the period of 6.4 cm and the maximum B field of 0.90 T, the gap can be varied in the range of the minimum 6 mm to the maximum 50 mm. (**a**) (left) Wavelength versus undulator period is plotted with the minimum gap (upper blue line) and the maximum gap (lower blue line). The wavelength in the range of [4.13 12.4] nm determines the optimum period of 6.4 cm. (**b**) (right) Modulator *K* parameter versus gap. The radiation wavelengths ([4.13 12.4]) nm corresponds to the gap between 20.9 and 8.5 mm and the *K* parameter between 3.5 and 6.4.

The EEHG beamline parameters are listed in Table 1. The turnabilities of the minimum and maximum wavelengths are set by the maximum and minimum allowed undulator gaps, respectively. To avoid the radiation damage, the maximum *B* field is limited to be less than $1.3 T$. For the radiator with the period of 6.4 cm and the maximum *B* field of $0.90 T$, the gap can be allowed to vary in the range of 6–50 mm. The vertical red line with arrow ends in Fig. 3a indicates the entire spectrum of the carbon K-edge. *K* parameter versus gap is shown in Fig. 3b. To cover the wavelength range of 4.13–12.4 nm, the gap needs to be varied in the range of 20.9 down to 8.5 mm, corresponding to *K* values of 3.50 and 6.38, respectively.

**Table 1:** The parameters of modulator 1, modulator 2, and radiator are listed, including period, total length, maximum *B* field, minimum, maximum, and operational gaps, and *K* parameter.

Eelement	Period	Length	unit	B_max_ (T)	Gap (mm)			Resonant wavelength (nm)			K (T∙cm)		
					min	max	operation	min	max	operation	min	max	operation
*MOD*_*1*_	*64*	256	cm	0.34			17.3			800			13.05
*MOD*_*2*_	*20*	100	cm	0.9			24.0			400			16.55
*Radiator*	*6.4*	352	cm	0.9	8.5	21	[8.5 21]	4.13	12.4	[4.13 12.4]	3.5	6.38	[3.5 6.38]

### Simulation for generating intense EUV and soft X-ray radiation

One expects that a smaller energy modulation becomes more critical for long undulators. A small energy modulation begins with a low energy spread as well as a small prebunching. The radiation power and the bunching are continuously growing through the undulator, eventually reaching the saturation power determined by the FEL Pierce parameter. Instead, a large energy modulation is often associated with a large prebunching, hence, allows a much faster growth of the CR power, even within a short undulator distance (e.g., a few meters). This is the case of the storage-ring-based EEHG FEL. The radiator length is limited to be ≤ 3.5 m in the NSLS-II.

The purpose of optimization becomes searching an optimal energy modulation, which provides the fastest growth of the CR power and simultaneously mitigates the effect of the rapid de-bunching induced by the large energy spread ($\sim 2 \cdot N_{u} \cdot \frac{{\Delta E_{tot} }}{E} \approx 2 \cdot \frac{{3.52\,{\text{m}}}}{{0.064\,{\text{m}}}} \cdot 2 \times 10^{ - 3} \approx 0.2$ for the nominal energy modulation). Here, $N_{u}$ refers to the number of undulator periods in the radiation stage. The optimal energy modulations depend on the harmonics; thus, we apply the optimization to the low (67) and high (183) harmonics. One expects that the optimal energy modulations of other harmonics should be bounded between the results of these two harmonics.

GENESIS simulations are applied to the optimization process^27^. The input parameter to the EEHG optimizer is the targeted harmonic number. Then, the beamline parameters, $A_{1,2}$ and $R_{2}$, are predicted by the EEHG optimizer. Other parameters, $\lambda_{1,2}$ and $R_{1}$, are fixed. A 6D phase space distribution with the longitudinally bunched and transversely matched beam profile are generated as the GENESIS input. The transverse profile of the electron beam is determined by the NSLS-II lattice parameters in the short straight section, as shown in Table 2.

**Table 2:** NSLS-II lattices and beam parameters include the Twiss at the short straight section and the RMS beam energy spread and normalized emittances.

Relative energy spread σ_E_/E	Beam energy E (MeV)	Energy spread σ_E_ (MeV)	x emittance ε_x_ (m rad)	y emittance ε_y_ (m rad)	I_peak_ (A)	β_x_ (m)	α_x_	β_y_ (m)	α_y_	Radiator period λ_u_ (cm)
0.001	3000	3	5.87 × 10^−6^	5.87 × 10^−8^	300	3.77	0	4.20	0	6.40

*Normalized emittances are used.

#### Simulation of harmonic 67

**Figure 4:**
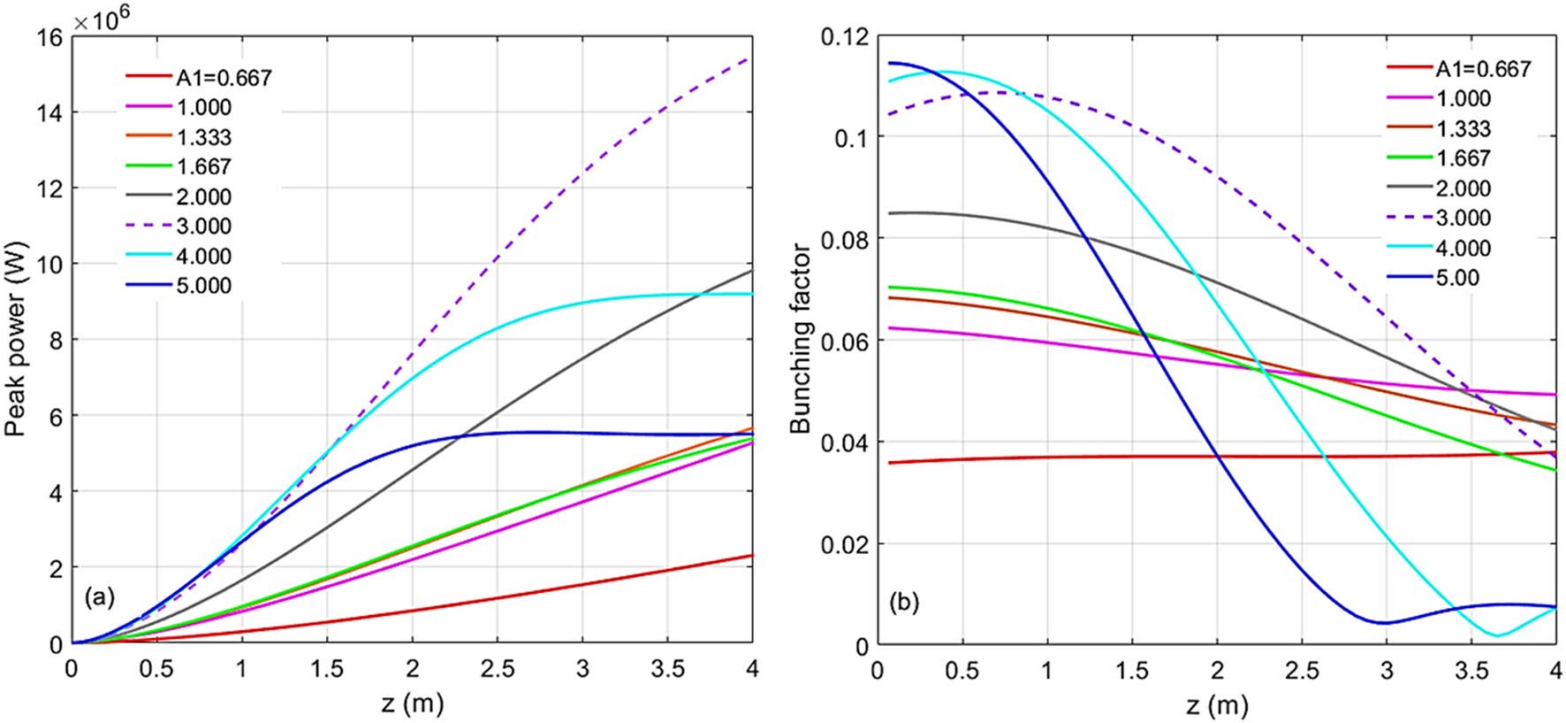
For the case of harmonic 67, the energy modulation of stage 1 is scanned with the values of 0.667 (red), 1.000 (magenta), 1.333 (orange), 1.667 (green), 2.000 (grey), 3.000 (purple), 4.000 (cyan), and 5.000 (blue). (**a**) Peak power versus undulator position. (**b**) Bunching factor versus undulator position.

**Figure 5:**
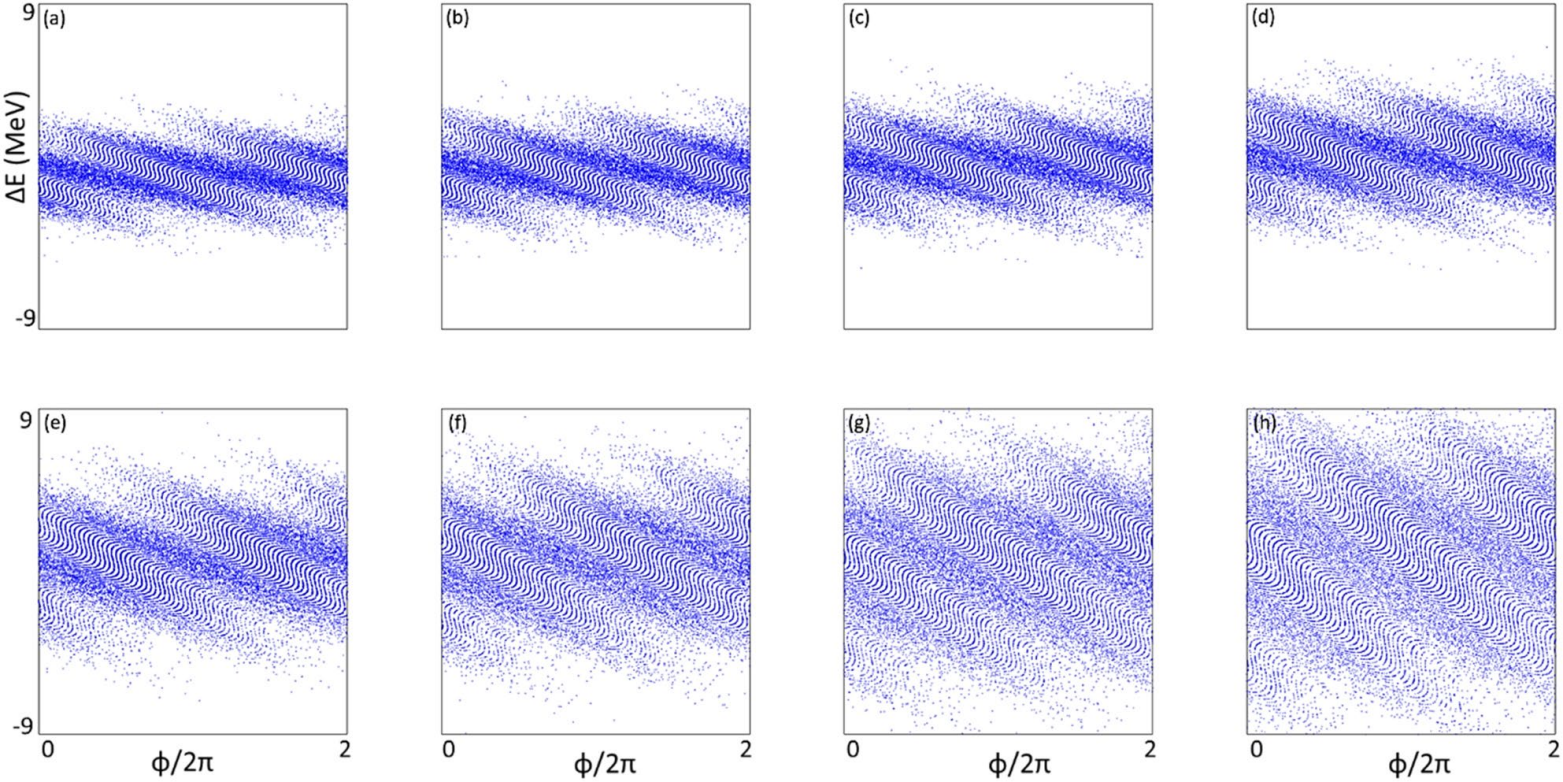
Longitudinal phase spaces corresponding to the energy modulations of stage 1 with the values of 0.667 (**a**), 1.000 (**b**), 1.333 (**c**), 1.667 (**d**), 2.000 (**e**), 3.000 (**f)**, 4.000 (**g**), and 5.000 (**h**) are plotted respectively.

**Figure 6:**
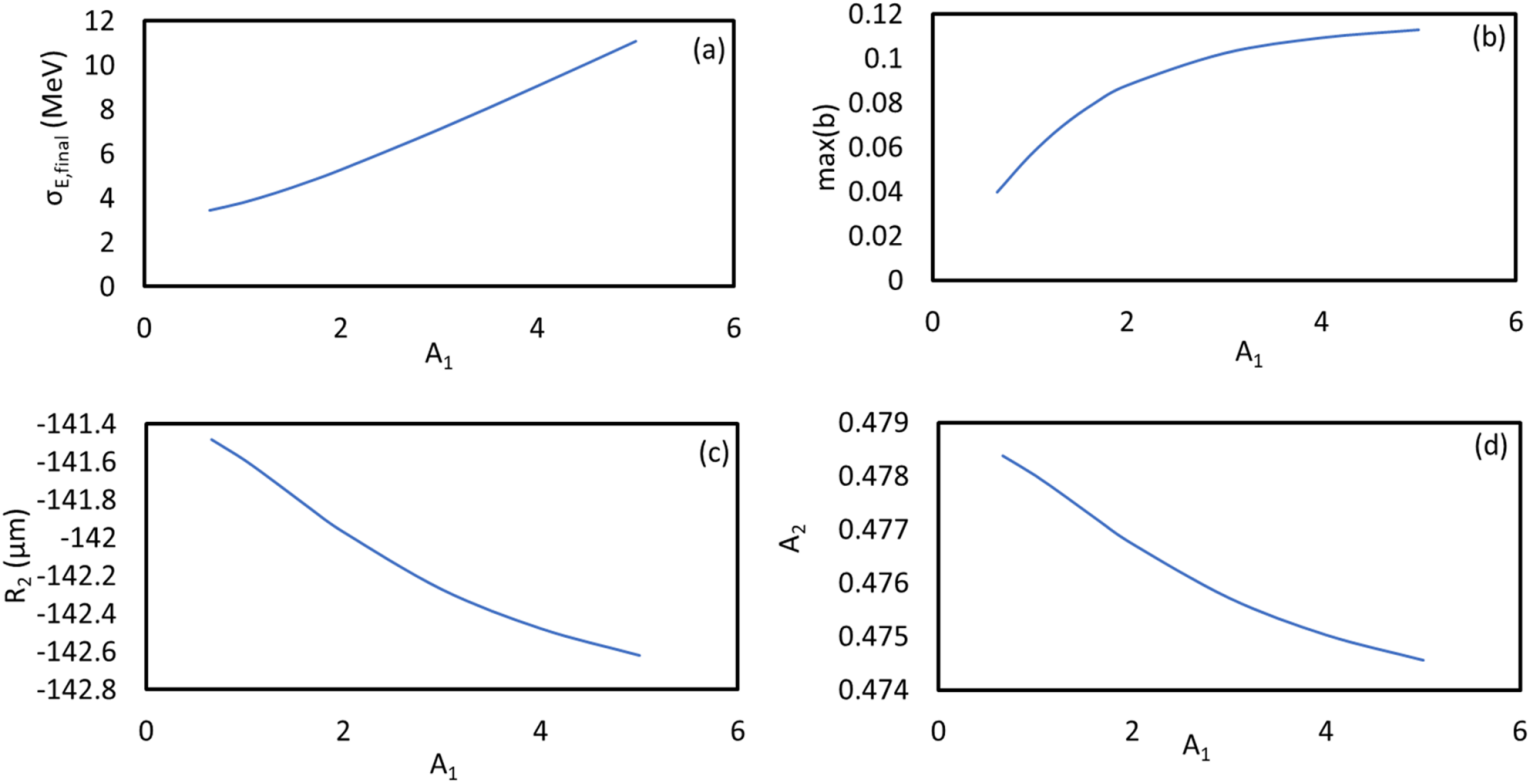
(**a**) The final effective RMS energy spread, (**b**) the maximal bunching, (**c**) the momentum compaction of chicane 2, and (**d**) the energy modulation of stage 2 as a function of the energy modulation of stage 1 are plotted, respectively.

For the case of harmonic 67, the scaled energy modulation of stage 1 has been varied from 0.667 to 5.000. The evolutions of the peak power and the bunching factor through the undulator are plotted in Fig. 4a and b, respectively. One extreme is to start with a large initial bunching but at the expense of the large energy spread induced fast de-bunching, hence, less CR gain at the exit of the undulator. The other extreme is to begin with a small initial bunching that slowly increases through the undulator. In-between these two cases, there is an optimal initial energy modulation of stage 1, which is around $A_{1} = 3.00$. The maximum bunching is achieved near the middle point of the undular length. Such optimized initial bunching allows a reasonably fast growth of the CR power as well as the bunching factor till the middle point of the undulator, then, the de-bunching process is mitigated and only mildly slows down the CR growth in the rest of the undulator, shown in Fig. 4a as the purple dashed line. The longitudinal phase spaces regarding these energy modulations of stage 1 with the values from 0.667 to 5.000 are shown in Fig. 5a–h, respectively. The final effective RMS energy spread, maximal bunching, momentum compaction of chicane 2, and energy modulation of stage 2 as a function of the energy modulation of stage 1 are plotted in Fig. 6a–d, respectively. From Fig. 6a, the optimal energy modulation $A_{1} = 3$ brings 130% increase of the energy spread for the modulated beam slice. We will discuss how this increased slice energy spread influences other beamline experiments in “Required mode for EEHG” section.

#### Simulation of harmonic 183

**Figure 7:**
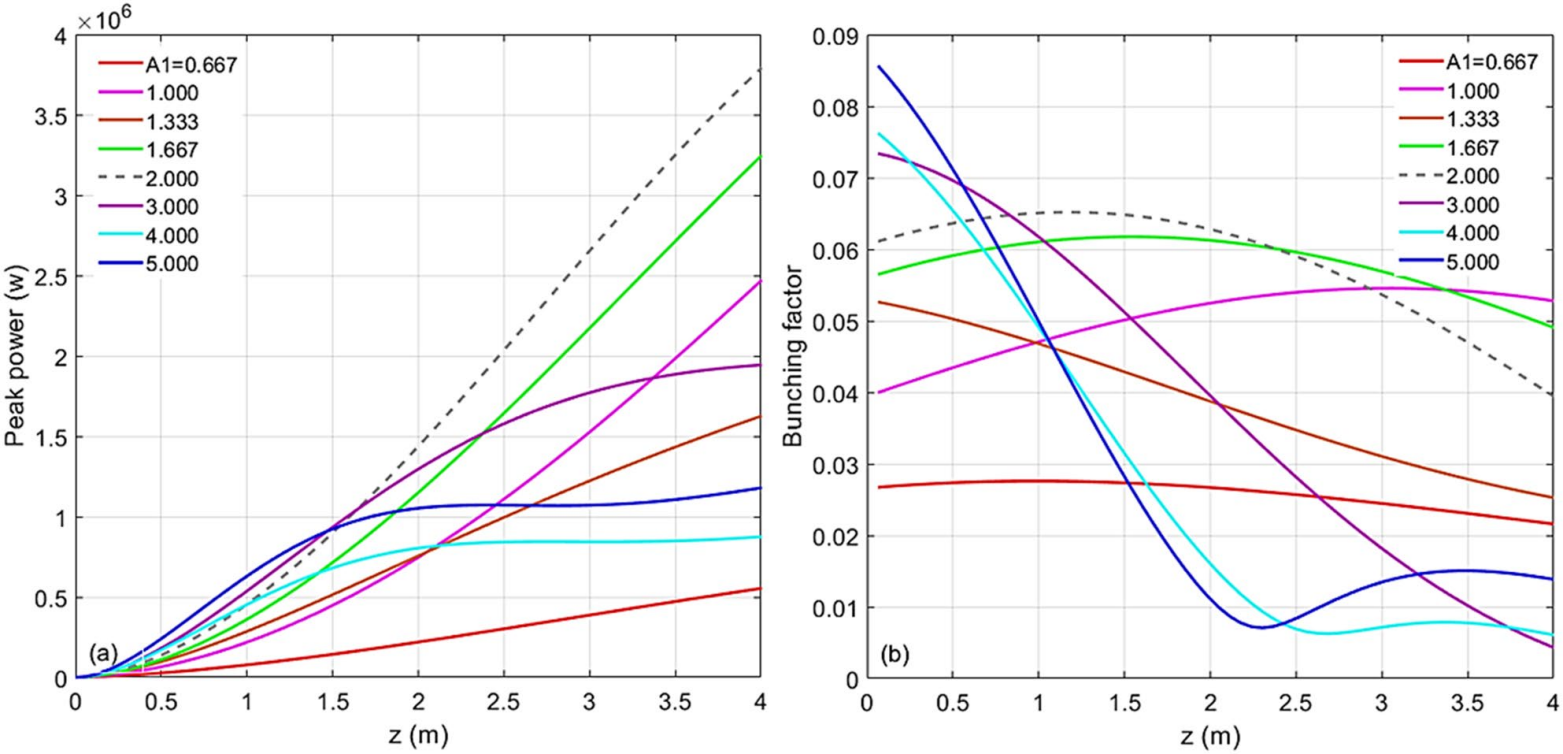
For the case of harmonic 183, the energy modulation of stage 1 is scanned with the values of 0.667 (red), 1.000 (magenta), 1.333 (orange), 1.667 (green), 2.000 (grey), 3.000 (purple), 4.000 (cyan), and 5.000 (blue). (**a**) Peak power versus undulator position. (**b**) Bunching versus undulator position.

**Figure 8:**
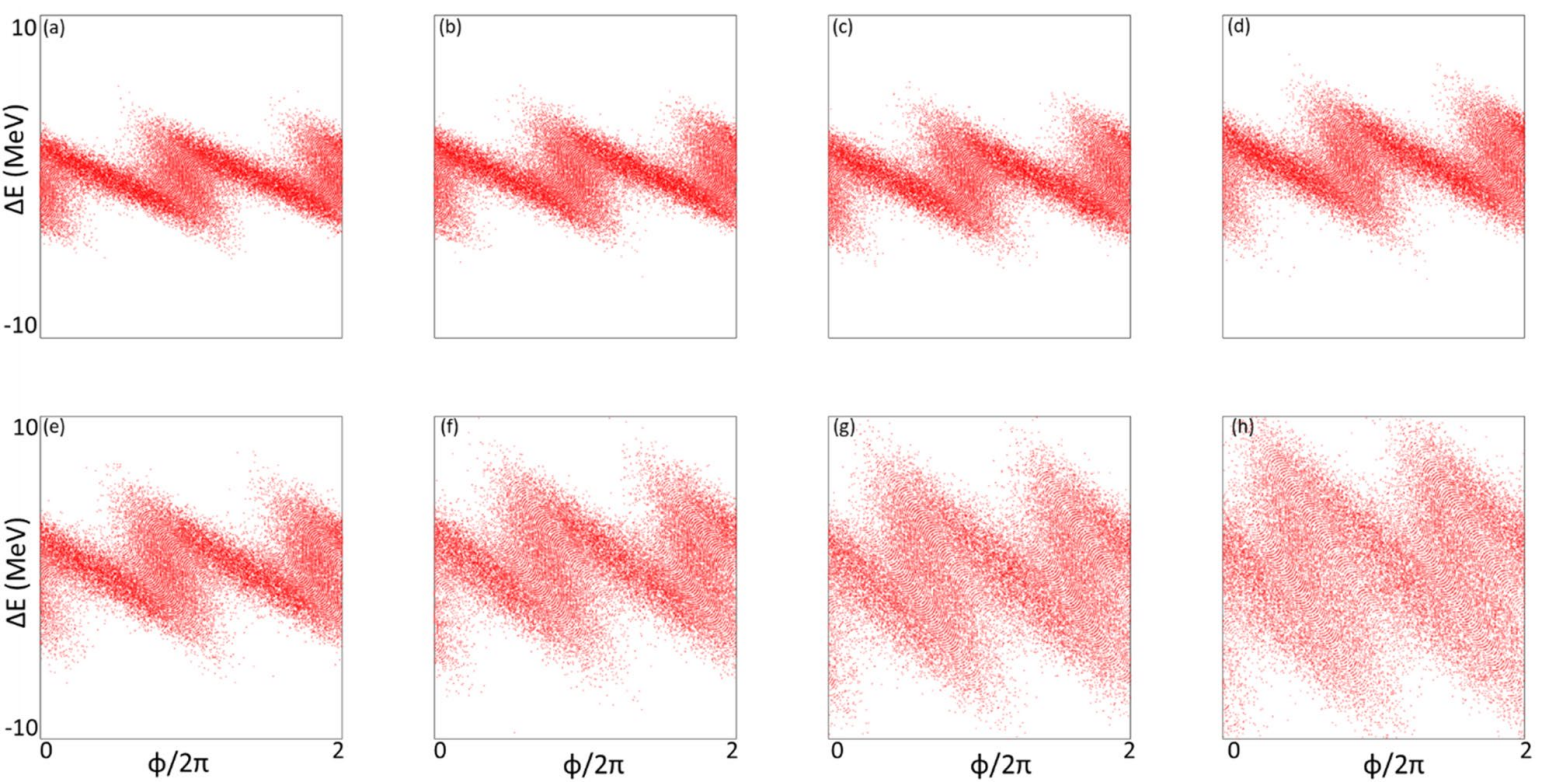
Longitudinal phase spaces corresponding to the energy modulations of stage 1 with the values of 0.667 (**a**), 1.000 (**b**), 1.333 (**c**), 1.667 (**d**), 2.000 (**e**), 3.000 (**f**), 4.000 (**g**), and 5.000 (**h**) are plotted.

**Figure 9:**
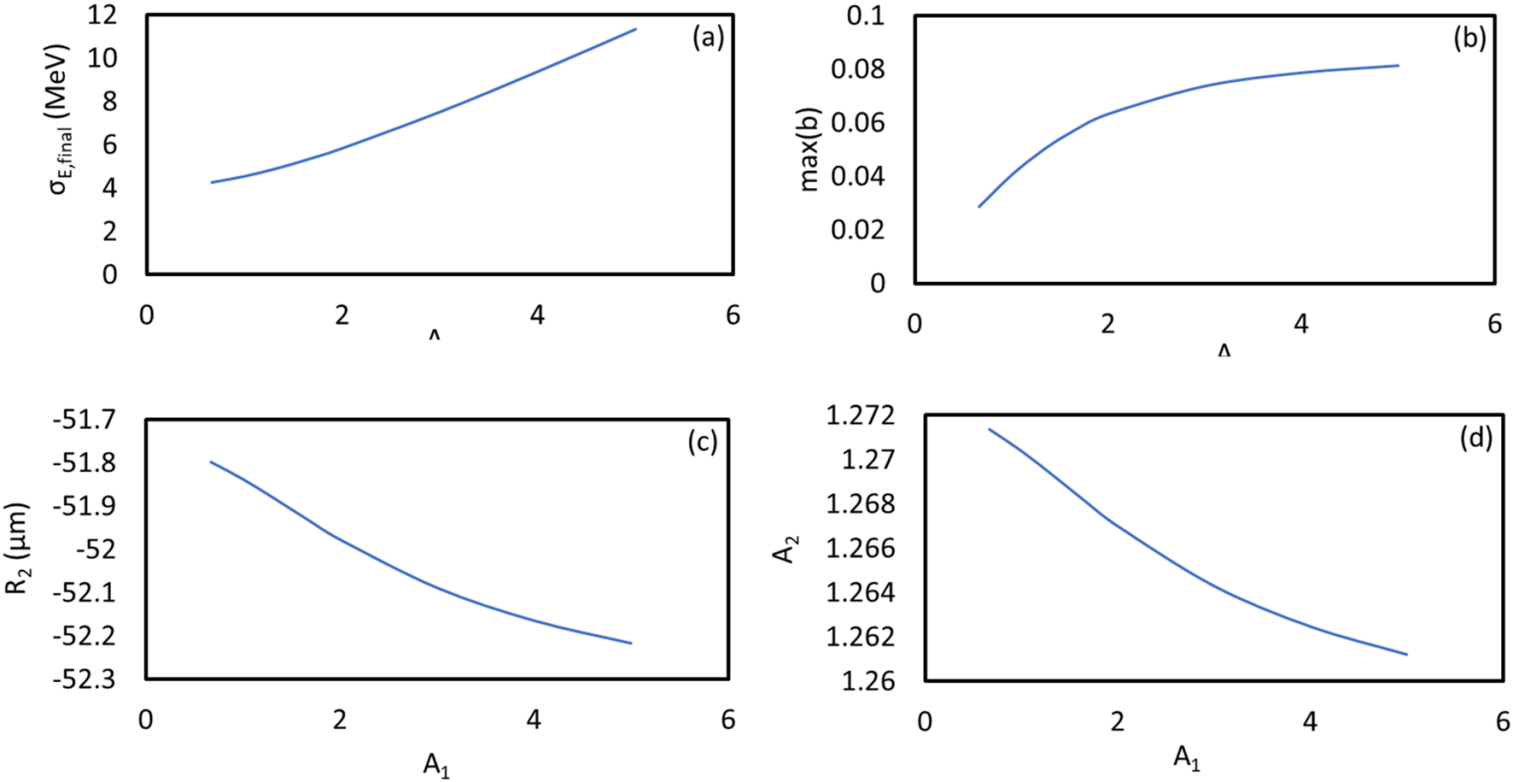
(**a**) The final effective RMS energy spread, (**b**) the maximal bunching, (**c**) the momentum compaction of chicane 2, and (**d**) the energy modulation of stage 2 as a function of the energy modulation of stage 1 are plotted, respectively.

Similarly, for the case of harmonic 183, the energy modulation of stage 1 has been varied among the values from 0.667 to 5.000. Peak power and prebunching evolving through the undulator distance are plotted in Fig. 7a and b, respectively. The longitudinal phase spaces corresponding to the energy modulations of stage 1 with the values from 0.667 to 5.000 are plotted in Fig. 8a–h, respectively. The final effective RMS energy spread, maximal bunching, momentum compaction of chicane 2, and energy modulation of stage 2 as a function of the energy modulation of stage 1 are plotted in Fig. 9a–d, respectively. The optimal energy modulation of stage 1 happens at $A_{1} = 2.00$, which causes 95% increase of the energy spread for the modulated beam slice. As one expects, shorter the radiator wavelength is, less tolerance the de-bunching process has.

#### Studies of energy spread induced de-bunching

**Figure 10:**
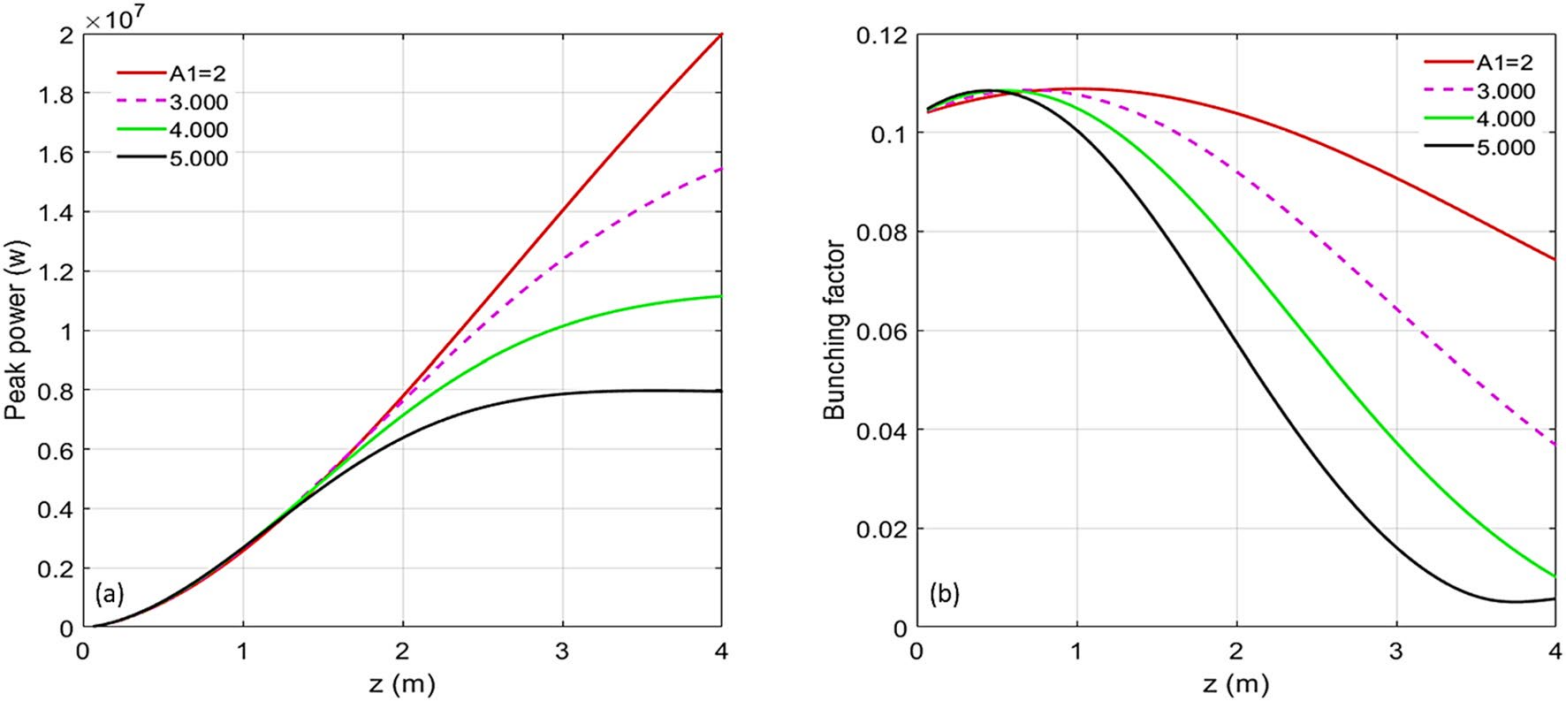
The energy modulation of stage 1 is scanned among the values of 2 (red), 3 (magenta), 4 (green), and 5 (grey) while the initial bunching is kept constant. (**a**) Peak power versus undulator position. (**b**) Bunching versus undulator position.

Usually, the energy modulation and the initial bunching factor are correlated. A higher energy modulation yields a higher initial bunching, but at the cost of a worse de-bunching effect. To solely study the energy spread induced de-bunching effect, we deliberately vary the effective energy spread while keeping the initial bunching constant. Larger initial energy spread causes faster de-bunching, which limits the CR power at the exit of the radiator. Higher harmonics usually require smaller energy modulation for a fixed undulator length. As one expects, the de-bunching becomes faster with the increase of the initial energy modulation. The optimal energy modulation happens while the maximum bunching is achieved in the middle point of the undulator length, shown as the red curves in Fig. 10a and b. Because the undulator is short and the initial bunching is substantial, FEL gain is not a concern and even higher harmonics can be produced until the de-bunching effect becomes strong for a length scale of about one meter.

#### Laser power and CR properties

The required powers of laser 1 and laser 2 as functions of the harmonic number and the energy modulation of stage 1 are shown as the contour plots in Fig. 1e and f, respectively. Those required maximal laser powers, a few GW and ten GW regarding laser 1 and 2, can be achieved with the current laser technologies.

The CR is estimated for the shorter and longer wavelengths of carbon K-edge, 4.37 nm, and 11.94 nm, including the peak power, the number of photons per pulse, and the RMS spectral bandwidth regarding three different pulse durations in RMS, 0.43 ps, 0.85 ps, and 1.23 ps. For each case, those output properties are estimated in two different radiator lengths, 2.5 m, and 3.5 m, as shown in Table 3.

**Table 3:** CR for the lower and upper wavelengths of carbon k-edge 4.37 nm and 11.94 nm, including the peak power, the number of photon pulse, and the RMS spectral bandwidth in three different pulse durations in RMS, 0.43 ps, 0.85 ps, and 1.23 ps are listed.

T_RMS_ (ps)	P_pk_ at *L*_*r*_ = 2.5 m (MW)		Photon per pulse at *L*_*r*_ = 2.5 m		P_pk_ at *L*_*r*_ = 3.5 m (MW)		Photon per pulse at *L*_*r*_ = 3.5 m		RMS bandwidth		Spectral brightness at 1 MHz	
	λ_67_ = 11.94 nm	λ_183_ = 4.37 nm	λ_67_ = 11.94 nm	λ_183_ = 4.37 nm	λ_67_ = 11.94 nm	λ_183_ = 4.37 nm	λ_67_ = 11.94 nm	λ_183_ = 4.37 nm	λ_67_ = 11.94 nm	λ_183_ = 4.37 nm	11.94 nm	4.37 nm
0.426	10.14	2.03	6.51 × 10^11^	4.77 × 10^10^	14.2	3.27	9.12 × 10^11^	7.69 × 10^10^	4.13 × 10^−5^	1.50 × 10^−5^	3.25 × 10^20^	7.54 × 10^19^
0.851			1.30 × 10^12^	9.54 × 10^10^			1.82 × 10^12^	1.54 × 10^11^	2.10 × 10^−5^	7.60 × 10^−6^	1.28 × 10^21^	2.98 × 10^20^
1.277			1.95 × 10^12^	1.43 × 10^11^			2.73 × 10^12^	2.31 × 10^11^	1.40 × 10^−5^	5.00 × 10^−6^	2.87 × 10^21^	6.78 × 10^20^

For each case, those output properties are calculated at two different radiator lengths, 2.5 m, and 3.5 m.

For the case of 3.5 m long radiator and 0.85 ps pulse duration, the maximum number of photons per pulse is 1.8 × 10^12^ regarding 11.9 nm. The repetition rate can be up to 1 MHz, hence, the correspond spectral brightness is 1.3 × 10^21^, which is more than two orders of magnitude higher than the current brightest source U (100) PMU (~ 1.1 × 10^19^) at the NSLS-II^28,29^.

#### Required mode for EEHG

The EEHG scheme requires that the NSLS-II storage ring is operated at an alternative mode with one hundred 5 mA electron bunches, equally spaced around the ring. Such a 5-mA electron bunch has the RMS bunch length > 20 ps, thus, the modulated slice only occupies a few percent of the entire bunch. Besides, the modulation only causes a local energy spread increase in the level of around 100%. One expects that the EEHG influences all other beamlines on the spectral brightness and the photon flux at the level of a few percent or less (this has been confirmed by Synchrotron Radiation Workshop simulations)^30^, hence, most beamlines can simultaneously operate in this EEHG mode.

## Method

### Implementing EEHG optimizer with ideal performance

After *m* is chosen (usually to be − 1)^8,9^, setting the parameters is comparatively straightforward. The EEHG process produces microbunching at many different wavelengths, but wavelengths corresponding to different *m* are either far apart or yield negligible bunching at nearby harmonics. Thus, only one *m* for a given optimized configuration needs to be considered.

The ideal bunching at a given wavelength is $b_{r} = {<\text{exp}}\left( { - ick_{r} t}\right)>$, and is given by

4 $$b_{r} = J_{p} \left( {k_{r} R_{2} \eta_{M2} } \right) \cdot J_{m} \left( {C\eta_{M1} } \right) \cdot exp\left( {\frac{ - 1}{2}C^{2} \sigma_{\eta }^{2} } \right),$$

where

5 $$C = k_{r} R_{2} + mk_{1} R_{1} .$$

$R_{1}$ and $R_{2}$ are the two momentum compactions, and $\eta_{M1}$ and $\eta_{M2}$ are the amplitudes of the two energy modulations. This assumes an initial Gaussian energy distribution.

When $\eta_{M1} \gg \sigma_{\eta }$, the bunching has an optimal value of $\left| {b_{r} } \right| = \left| {J_{p} \left( { \pm j_{p,1}^{^{\prime}} } \right)J_{m} \left( { \pm \hat{j}_{m,1} } \right)} \right|$, where the arguments of the Bessel functions give the maximum values. Defining $A_{1,2} = \eta_{M1,2} /\sigma_{\eta }$, when $A_{1}$ is reduced and the exponential damping terms becomes relevant, the optimum bunch is given by

6 $$\left| {b_{r} } \right| = \left| {J_{p} \left( { \pm j_{p,1}^{^{\prime}} } \right)J_{m} \left( { \pm \hat{j}} \right)exp\left( {\frac{ - 1}{2}\frac{{\hat{j}^{2} }}{{A_{1}^{2} }}} \right)} \right|,$$

where $j_{p,1}^{^{\prime}}$ is the location of the first maximum of the Bessel function $J_{p}$. A rough estimate for $\hat{j}$ is given by

7 $$\hat{j} \cong \frac{{j_{m,1}^{^{\prime}} }}{{1 + \frac{{\sigma_{\eta }^{2} }}{{\eta_{M1}^{2} }}\left[ {1 - \left( {\frac{m}{{j_{m,1}^{^{\prime}} }}} \right)^{2} } \right]^{ - 1} }}.$$

A good asymptotic expansion for large values of *p* is

8 $$j_{p,1}^{^{\prime}} \cong p + 0.80861 \cdot p^{1/3} + 0.07249 \cdot p^{ - 1/3} - 0.05097 \cdot p^{ - 1} .$$

For the lowest indices (e.g., $p \le 10$), it is better to just use a table of values for $j_{p,1}^{^{\prime}}$, and the optimal settings can also be calculated directly.

The final energy spread can approximately be given by

9 $$\sigma_{\eta f}^{2} = \sigma_{\eta }^{2} + \frac{1}{2}A_{1}^{2} + \frac{1}{2}A_{2}^{2} .$$

To make EEHG effective, the value of *C* should be much smaller than the two terms $k_{r} R_{2}$ and $k_{1} R_{1}$ (see Fig. 1c in “Appling optimizer to NSLS-II” section). This is equivalent to requiring that $\eta_{M1} k_{1} R_{1} \gg 1$. There are two distinct solutions that generate the same bunching, since *C* can either be positive or negative, and either choice yields the same bunching (see Fig. 1i in “Appling optimizer to NSLS-II” section). This is true even when all dispersive sections have the normal sign for $R_{56}$. Comparing the form of optimization, we find $C = \pm \frac{{\hat{j}}}{{\eta_{M1} }}$, which implies

10 $$\begin{aligned} R_{2} & = \frac{{C - mk_{1} R_{1} }}{{k_{r} }} = \frac{{ \pm \frac{{\hat{j}}}{{\eta_{M1} }} - mk_{1} R_{1} }}{{k_{r} }}, \\ \eta_{M2} & = \frac{{j_{p,1}^{^{\prime}} }}{{k_{r} \cdot R_{2} }}. \\ \end{aligned}$$

The two optimal solutions have slightly different values for $R_{2}$ and $A_{2}$. The first term in $R_{2}$ is going to be small, so $R_{2} \approx - \frac{{mk_{1} R_{1} }}{{k_{r} }};$ however, such small correction cannot be ignored when evaluating *C*.

Based on these procedures, we have implemented EEHG optimizer for tuning all important parameters to achieve the ideal performance of an EEHG beamline in a synchrotron light source.

## Discussion

The EEHG seeding option could offer very narrow bandwidths and extremely high brightness, realized by diffraction-limited short pulses in transverse planes and Fourier-limited bandwidth in the EUV to soft X-ray spectrum. The attractive FEL features are negligible heat load on the beamline optics due to the narrow bandwidth and a potential capability to generate short pulses using a short pulse seeding. The advantage of a storage ring FEL compared to a linac-based one is much better pulse-to-pulse beam stability.

For the storage-ring-based EEHG FELs, we have implemented a complete set of tools, named EEHG optimizer, which can provide the optimized parameters for generating the longitudinal bunched and transverse matched 6D phase space distribution. Then, GENESIS simulation is applied to search the optimal energy modulation regarding each specific harmonic, aiming the highest CR gain as well as mitigated energy-spread induced de-bunching. This toolkit has been successfully applied to the NSLS-II storage ring as an example, with an up to two orders of magnitude improvement of the spectral brightness for the wavelength of 12 nm. Furthermore, the EEHG scheme can expand the capability of the NSLS-II with a fully coherent time-resolved tunable EUV and soft X-ray radiation source, also, such scheme is compatible with other beamlines with the minimum impact of NSLS-II routine operation. Compared to other storage-ring-based FELs, e.g., angular dispersion enhanced prebunching scheme for seeding coherent EUV and soft X-ray FEL^6,7,31–35^, the EEHG approach holds a great promise, not only for its simplicity (no need of any lattice change) but also for the accessibility to much higher harmonics, toward the soft X-ray spectrum. This is because for the EEHG approach^8,9^, the maximal bunching (*b*_*n*_) decreases with the harmonic *n* as $n^{{\frac{ - 1}{3}}}$, as shown in Fig. 1g; instead, for the angular dispersion enhanced prebunching scheme^7^, like HGHG, *b*_*n*_ decreases much faster with *n*, as *n*^−1^. Thanks to our compact design, modulator 2 (1 m), chicane 2 (< 0.5 m), and radiator (3.5 m) can all be fit into the 5 m short straight section.

Since the toolkit is designed and generalized to any type of a synchrotron light source based EEGH beamline, it can be easily extended to the 4th generation storage rings with the expected much better performances because of the diffraction-limited ultrasmall emittances.

There are usually three types of effects, which can impact the performance of an EEHG FEL. Wake fields and noise in the laser peak power can disrupt the coherence of the output radiation pulse, although they should not influence the total power produced. Incoherent energy scattering can reduce the bunching as well as the output power, but for the parameters used here, this should not be a concern, in particular because of the large amplitude of the energy modulations required by the bunching process. For a storage ring, however, there is the additional possibility of collective effects disrupting the electron beam after many turns around the ring.

A combination of the high bunch intensity and perturbations of the bunch over short scale lengths can result in strong collective effects, which may affect the electron bunch parameters. Beam interaction with the machine impedance could lead to the bunch lengthening and excitation of the microwave instability, which increases the energy spread. The bunch slicing will enhance the effects of coherent synchrotron radiation (CSR) including 3D emittance growth and possible excitation of the CSR burst instability. Quantitative analysis of the collective effects requires further investigation including extensive numerical simulations using specific parameters of the ring lattice, light-generating insertion devices, and vacuum chamber.
